# Seroprevalence and comparison of different serological tests for brucellosis detection in small ruminants

**DOI:** 10.14202/vetworld.2015.561-566

**Published:** 2015-05-04

**Authors:** Dashrath B. Sadhu, H. H. Panchasara, H. C. Chauhan, D. R. Sutariya, V. L. Parmar, H. B. Prajapati

**Affiliations:** 1 Department of Veterinary Medicine, Livestock Research Station, Sardarkrushinagar Dantiwada Agricultural University, Sardarkrushinagar, Dantiwada, Gujarat, India; 2 Department of Animal Biotechnology, College of Veterinary Science & Animal Husbandry, Sardarkrushinagar Dantiwada Agricultural University, Sardarkrushinagar, Dantiwada, Gujarat, India

**Keywords:** brucellosis, seroprevalence, serological test, small ruminant

## Abstract

**Aim:**

The aim was to study the seroprevalence and efficacy of the different serological tests used for detection of antibody against *Brucella* species in small ruminants of Banaskantha district of North-Gujarat.

**Materials and Methods:**

Total 1000 serum samples comprising of 485 from sheep and 515 from goat tested for detection of antibodies against the *Brucella* species by three different serological tests *viz*., Rose bengal plate test (RBPT), Standard tube agglutination test (STAT), and Indirect Enzyme-linked immunosorbent assay (I-ELISA).

**Results:**

The seroprevalence of brucellosis in small ruminants was 11.30%, 11.10%, and 8.80% by RBPT, STAT, and I-ELISA, respectively. The seroprevalence of brucellosis was found to be higher in sheep than goats. The sensitivity of RBPT was found slight more than STAT, but the specificity of both tests was same. In this study, the overall agreement of RBPT and STAT with I-ELISA was found 92.50% and 92.30% in small ruminants, respectively.

**Conclusion:**

I-ELISA was a better serological test as compared to RBPT and STAT in the sense of sensitivity, specificity, and rapidity and it could be advocated for screening of brucellosis in sheep and goats.

## Introduction

### Brucella is a Gram-negative facultative intracellular organism responsible for a variety of disease conditions and having zoonotic significance. Brucellosis is caused by bacteria of the genus Brucella and is reported worldwide causing abortion, infertility, retained placenta, endometritis in females and to a smaller extent, orchitis, and infection of the accessory sex glands in males [1].

Brucellosis in India is a very common but often neglected disease [2]. Brucellosis due to *B. melitensis* is widespread in India and major cause of abortion in small ruminants imposing economic loss due to an adverse effect on total animal protein supplies and severe hazard to human health [3,4]. Sheep brucellosis can be divided into classical brucellosis and ram epididymitis. Ram epididymitis is caused by non-zoonotic agent *B. ovis*, while classical brucellosis is caused by *B. melitensis* and constitutes a major public health threat equal to goat brucellosis [5]. There are about 500,000 new human cases of brucellosis reported annually worldwide making it the most common zoonosis [6]. Sulima and Venkataraman [7] calculated the average annual economic loss due to brucellosis per animal on sheep was found to be INR. 1180/- and on goats INR. 2121.82.

Brucellosis appears to be increase in recent times, perhaps due to increased trade and rapid movement of livestock [2]. Free grazing and movement with frequent mixing of flocks of sheep and goats are the main mode of disease transmission resulting in high prevalence and wide distribution of brucellosis in these animals in India [8].

Status of the diseases in small ruminants in the country can be known only through effective sero-monitoring using serological tests and random sampling methods for the disease. The economic importance of brucellosis in sheep and goats requires the use of sensitive and rapid diagnostic methods. Diagnosis of *B. ovis* and *B. melintensis* infection is based on clinical examination, serological tests, biotechnological techniques, and isolation [9,10]. The laboratory isolation and identification of *Brucella* organisms are most reliable methods of diagnosis but are not successful always, is not practicable in terms of time and labor for field and laboratory personnel when large numbers of animals are involved and also cumbersome and pose great risk to the laboratory personnel. The biotechnological procedures require the establishment of advanced laboratories and trained persons.

Rose bengal plate test (RBPT) is simple and easy to perform and can be used as herd screening test at remote places. Joint FAO/WHO Expert Committee on Brucellosis recommended the use of RBPT as a useful screening test for diagnosis of *B. melitensis* infection in sheep and goats [11]. Standard tube agglutination test (STAT) quantifies total agglutinating antibodies and higher detection rates had been reported through STAT in sheep and goat [12]. Recently, Enzyme-linked immunosorbent assay (ELISA) has taken over as an important serological tool in the diagnosis of brucellosis because of its economy, sensitivity, specificity, rapidity, reproducibility, and easy interpretation through colorimetric end product [13]. Enzyme immunoassays are superior to all other tests in terms of specificity and sensitivity [12]. Due to non-availability of a gold standard test except isolation of organism which is a cumbersome process the improvement and validation of available serological tests is a needed as it enhances the specificity and sensitivity of the test.

Small ruminants play a significant role in supporting the livelihood system of the rural poorest men and women. More than 98% of sheep and goats are owned by the small, marginal, and landless illiterate farmers of the villages. They are unaware about brucellosis disease. Gujarat state is known for its rich biodiversity in most of the livestock species. Banaskantha district has 309 thousand goats and 161 thousand sheep population [14]. Goat and sheep-rearing continues to be a backward profession and thus has acquired very less attention. Thus, the aim of the present study was sero-screening of brucellosis in the cases of abortion and various reproductive disorders in sheep and goats by using three serological tests *viz*., RBPT, STAT, and indirect -ELISA and to evaluate their comparative efficacy.

## Materials and Methods

### Ethical approval

This study was approved by Animal Ethics Com­mittee of Sardarkrushinagar Dantiwada Agricultural University

### Study area

The seroprevalence of brucellosis carried out in small ruminants of Banaskantha district of Gujarat, India. Banaskantha district is situated in Northwestern part of the Gujarat. The district is encompassed by 23.03-24.45 North latitude and 71.21-73.02 East longitude. The district is surrounded by Rajasthan state in East-North, Mehsana in South and Patan and Kutch district in West. Next to the desert is the border of Pakistan. The normal climate of the district and mainly three seasons *viz*. summer, monsoon, and winter. The normal rainfall of the district could be considered at 601 mm. It is the second largest district in the state.

### Samples

Total 1000 serum samples of small ruminants comprising 485 from sheep and 515 from goats, having the history of abortion and reproductive disorders like endometritis, retention of placenta, infertility and repeat breeding, were randomly collected from different 17 locations of six taluka *viz*., Bhabhar, Dantiwada, Deesa, Deodar, Dhanera and Palanpur of Banaskantha district of North Gujarat during March, 2013 to March, 2014. The samples were stored at −20°C until they were used. All the serum samples tested for the presence of *Brucella* antibodies by using three serological tests *viz*., RBPT, STAT, and I-ELISA.

### RBPT protocol

**Figure-1:**
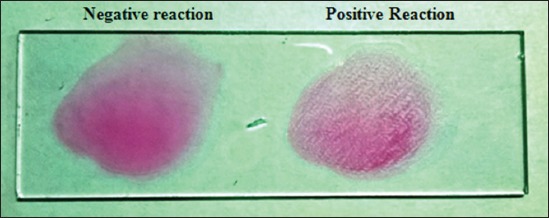
Rose Bengal plate test.

The RBPT was performed as per the procedure described by Alton *et al*. [15]. The RBPT antigen was procured from the Institute of Animal Health and Veterinary Biologicals (IAH and VB), Hebbal, Bangalore, Karnataka, India. To perform the test, antigen and serum were brought to the room temperature. The bottle containing antigen was shaken well to ensure homogenous suspension. Then after, one drop (0.03 ml) of serum sample and antigen was taken on the same slide using different micropipette and mixed thoroughly using a spreader. The slide was rotated for 4 min and observed immediately then after 4 min for results. A result was considered as positive when there was noticeable agglutination found after 4 min for results (Figure-1).

### Stat protocol

**Figure-2:**
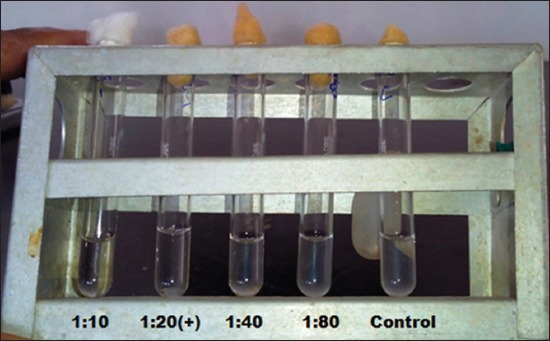
Serum tube agglutination test, Tube No. 1, 2=Positive reaction, Tube No. 3, 4=Negative reaction, Tube C = Control.

The STAT was performed as per the method described by Alton *et al*. [15]. The *Brucella abortus* plain antigen was procured from Institute of Animal Health and Veterinary Biologicals (IAH and VB), Hebbal, Bangalore, Karnataka, India. In order to perform the test, 0.8 ml of 0.5% phenol saline was taken in the first agglutination tube whereas 0.5 ml of the same was taken in remaining four agglutination tubes placed in a rack. Then after 0.2 ml of serum sample was added in the first tube and mixed well by shaking. The 0.5 ml of diluted serum was transferred from first to the second tube and the process was repeated up to the fifth tube. The 0.5 ml of diluted serum was discarded from the last tube and 0.5 ml of *Brucella abotus* plain antigen was added to each tube to get final dilution of 1:10, 1:20, 1:40, 1:80, and 1:160 in first, second, three, four, and fifth tube, respectively. A control tube was set up to simulate 50% agglutination by mixing 0.5 ml antigen and 1.5 ml of 0.5% phenol saline in an agglutination tube. All six tubes incubated at 37°C for 20 h before observation. Sera samples showing agglutination at 1:20 (40 IU) titer per ml of serum or above was considered as to be positive (Figure-2).

### I-ELISA protocol

**Figure-3:**
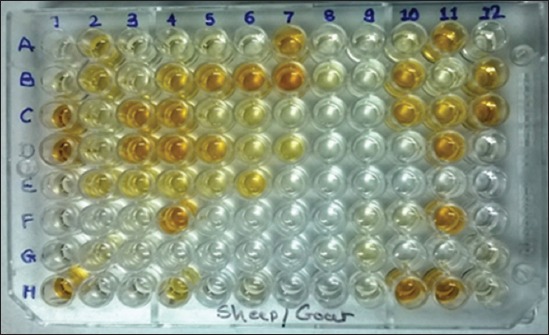
Microtiter plate showing the results of I-ELISA, Well A1 and B1- Conjugate control, Well C1 and D1 - Strong positive control, Well E1 and F1 - Moderately positive control, Well indicate positive reaction of field sera - H1, B2, C3, D3, B4, C4, D4, F4, B5, D5, B6, A7, B7, B10, C10, A11, C11, D11, F11, H11, B12, and C12, rest of well - indicate negative reaction.


The I-ELISA was performed using the Protein-G based kit for Caprine and Ovine Brucellosis manufactured by Project Directorate on Animal Disease Monitoring and Surveillance, Hebbal, Bangalore, Karnataka, India using smooth lipopolysaccharide for coating. In order to perform the test, samples, reagents, and plate(s) were brought to room temperature prior to starting the test. Using a pipette set at 100 μl, antigen was transferred into each well of the microtiter plate. The sides of the plate were tapped to ensure even distribution of the antigen over the bottom of each well. The plate was covered with aluminum foil/lid and incubated overnight at 4°C in the refrigerator. After incubation, the plate was washed with 100 ml of washing buffer. For manual washing, the contents of the wells were dumped into a sink and the free content was removed by striking. The plate inverted on a clean paper towel for 4 times. Immediately, the wells were filled with washing buffer using a multichannel pipette and the washing procedure was repeated for 2 times. The diluted 100 µl test sera sample was dispensed in duplicate wells (two-wells) and three control sera (High, moderate, and negative sera) in quadruplicate wells (four-wells) of the microtiter plate as per the layout provided and incubated at 37°C for 1 h on the ELISA plate shaker @ 300 rpm. The plate was removed from the shaker and washed three times with washing buffer. Then 100 µl of working dilution of chromogen solution was added to each wells of the microtiter plate and incubated at room temperature in dark place for 7 min or until a visible color developed in the strong positive wells by covering with aluminum foil. Immediately after the color developed, further reaction was stopped by adding 50 µl of stopping solution to each well of the microtiter plate. Immediately after adding the stopping solution, the plate was read in the ELISA plate reader at 492 nm wavelength (Figure-3). The optical density (OD) values of the test controls in each ELISA test performed should fall within the defined upper control limit (UCL) and lower control limit (LCL) for acceptance and rejection of the test. For positive control, OD values of UCL, and LCL should be between 1.2 and 0.06, respectively. While for the negative controls, OD values of UCL, and LCL should be between 0.20 and 0.09, respectively. Percent positivity (PP) value was calculated as follows:

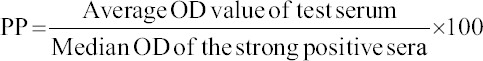


Sample that gave more than 55% PP value was considered as positive, below 55% was considered as negative if sample showed PP value 55% then it was retested.

### Statistical analysis

A Chi-square (*χ*^2^) test was done to compare the prevalence of brucellosis (in percent) between species. Significance was determined at 5% level. The difference was considered statistically significant if the p<0.05. The comparative efficacy of RBPT and STAT to I-ELISA determined with regards to their sensitivity, specificity, and overall agreement in the diagnosis of small ruminant brucellosis. Cross tabulation of RBPT and STAT with I-ELISA, considering I-ELISA as a gold standard test were recorded as per Samad *et al*. [16] to determine relative sensitivity, specificity, and overall agreement of RBPT and STAT.

Sensitivity: It is the capacity of the test to detect diseased animals, when compared with the gold standard test (a/a+c ×100).

Specificity: It is the capacity of the test to detect non-diseased animals, when compared with the gold standard test (d/b+d × 100).

Overall agreement: Is the proportional similarity of the results of both the tests (a+d/N × 100).

## Results and Discussion

### Seroprevalence

The overall seroprevalence of brucellosis in small ruminants was 11.30%, 11.10%, and 8.80% by RBPT, STAT, and I-ELISA, respectively. Statistically significant (p<0.05) higher seroprevalence was found in sheep (14.64%, 14.43%, and 11.75%) than in goats (8.15%, 7.96%, and 6.02%) by RBPT, STAT, and I-ELISA test, respectively (Table-1).

**Table 1:** Comparison of all three serological test with respect to species.

Species	Number of sera sample tested	RBPT positive (%)	STAT positive (%)	IELISA positive (%)
Goats	515	42 (8.15)	41 (7.96)	31 (6.02)
Sheep	485	71 (14.64)	70 (14.43)	57 (11.75)
Total	1000	113 (11.30)	111 (11.10)	88 (8.80)
*χ*^2^ test (p value)		10.48* (p=0.002)	10.60* (p=0.001)	10.23* (p=0.002)

* Significant at 5% Level (p<0.05), RBPT=Rose Bengal plate test, STAT=Standard tube agglutination test, I-ELISA=Indirect enzyme-linked immunosorbent assay

Earlier, similar seroprevalence of brucellosis was reported by Shome *et al*. [17] 9.95% by RBPT and 7.36% by I-ELISA in sheep and goats of Rajasthan, Gujarat, and Karnataka state and Singh *et al*. [18] reported higher prevalence of brucellosis in ovine species as compared to caprine species by multiple serological tests.

Teshale *et al*. [19] reported 9.7% positivity by I-ELISA in sheep and goats of pastoral regions of Ethiopia. Ashenafi *et al*. [20] reported 9.4% positive samples by RBPT in sheep and goats of the pastoral region of Afar. In comparison to the present study high seroprevalence was obtained by Esendal *et al*. [21], who reported 37.6% and 44.4% by RBPT and STAT, respectively, while 26.99% by I-ELISA according to Valarmathy *et al*. [22] in ovine-caprine sera. The overall prevalence of brucellosis in sheep of Kashmir valley was 6.50% [23]. The reason for this seroprevalence in small ruminants could be variation in management practices, frequent introduction of new animals without proper serological testing, no any practice of isolation, and removal of animals with high incidence of abortions, frequent mixing of one infected flock to another, mixing of different species, no any proper disposals of aborted fetus and placental membranes, and contamination of healthy animal to contaminated feed and water.

In the present study, when three serological tests were compared, highest seropositivity was found by RBPT (11.30%) followed by STAT (11.10%) and least by I-ELISA (8.80%). Similar results noted by Rahman *et al*. [24] who found highest seroprevalence of brucellosis by RBPT (5.83%) followed by STAT (4.17%) and least by I-ELISA (2.50%) in goats and highest seroprevalence of brucellosis by RBPT (3.75%) followed by STAT (2.50%) and least by I-ELISA (1.25%) in sheep. Din *et al*. [25] also found RBPT (11.33%) was more sensitive than SPAT (9.33%) and STAT (7.66%) in goats. However, Kotadiya [26] reported higher seroprevalence by I-ELISA (18.20%) followed by RBPT (11.38%) and least by STAT (9.44%) in sheep of Gujarat. Shome *et al*. [17] reported highest seroprevalence of brucellosis by RBPT (9.95%) followed by I-ELISA (7.36%) and least by STAT (5.67%) in sheep and goats of Rajasthan, Gujarat, and Karnataka states of the India. It may be due to the ability of each test to detect different antibody classes. The I-ELISA detected least positivity of brucellosis while RBPT detected higher positivity of brucellosis in small ruminant. It could be due to I-ELISA is a quantitative test which detects only IgG while STAT quantifies both IgM and IgG (but mainly IgM) and RBT qualitatively detects both IgM and IgG. Beside this, RBPT was highly sensitive but heterospecific. Infection due to organisms such as *Vibrio cholera*, *Yersinia enteroclitica* 0:9, *Pasteurella* spp, *Salmonella* or some other members of the *Brucellaceae* family could give false positive results in RBPT than STAT and I-ELISA.

### Sensitivity, specificity, and overall agreement

In the present study, the sensitivity of RBPT and STAT was 71.59% and 69.32%, respectively while specificity was 94.52% for both the tests, considering I-ELISA as a gold standard test. Thus, RBPT was more sensitive than STAT but the specificity of both tests was similar (Table-2). Similar results also obtained in caprine and ovine species individually (Tables-3 and 4).

**Table 2:** Sensitivity, specificity, and overall agreement of RBPT and stat in comparison to I-ELISA for detection of *Brucella* antibodies in small ruminants.

Test	I-ELISA		Total	Sensitivity (%)	Specificity (%)	Overall agreement (%)

	Positive	Negative				
RBPT						
Positive	63	50	113	71.59	94.52	92.50
Negative	25	862	887			
Total	88	912	1000			
STAT						
Positive	61	50	111	69.32	94.52	92.30
Negative	27	862	889			
Total	88	912	1000			

RBPT=Rose Bengal plate test, STAT=Standard tube agglutination test, I-ELISA=Indirect enzyme-linked immunosorbent assay

**Table 3:** Sensitivity, specificity, and overall agreement of RBPT and stat in comparison to I-ELISA for detection of *Brucella* antibodies in goat.

Test	I-ELISA		Total	Sensitivity (%)	Specificity (%)	Overall agreement (%)

	Positive	Negative				
RBPT						
Positive	19	23	42	61.29	95.25	93.20
Negative	12	461	473			
Total	31	484	515			
STAT						
Positive	18	23	41	58.06	95.25	93.01
Negative	13	461	474			
Total	31	484	515			

RBPT=Rose Bengal plate test, STAT=Standard tube agglutination test, I-ELISA=Indirect enzyme-linked immunosorbent assay

**Table 4:** Sensitivity, specificity, and overall agreement of RBPT and stat in comparison to I-ELISA for detection of *Brucella* antibodies in sheep.

Test	I-ELISA		Total	Sensitivity (%)	Specificity (%)	Overall agreement (%)

	Positive	Negative				
RBPT						
Positive	44	27	71	77.19	93.69	91.75
Negative	13	401	414			
Total	57	428	485			
STAT						
Positive	43	27	70	75.44	93.69	91.55
Negative	14	401	415			
Total	57	428	485			

RBPT=Rose Bengal plate test, STAT=Standard tube agglutination test, I-ELISA=Indirect enzyme-linked immunosorbent assay

Similarly, Tayshete [27] reported 71.42% sensitivity of both RBPT and STAT and 100% specificity of both RBPT and STAT in small ruminants, considering I-ELISA as a gold standard test. Awandkar *et al*. [28] noted that RBPT was more sensitive than STAT. In contrast, Hassanain and Ahmed [29] reported STAT (100%) was more sensitive than RBPT (83.3%) for diagnosis of brucellosis. Hence, I-ELISA has been found to detect antibodies in chronically infected animals while RBPT detects antibodies only in acutely infected animals and it is high sensitive and specific for antibody detection; this method has been recommended to be stable and suitable test for routine diagnosis of brucellosis in small ruminants.

The overall agreement of RBPT and STAT with I-ELISA was found 92.50% and 92.30% in small ruminants, respectively (Table-2). Similar results also obtained in caprine and ovine species individually (Tables-3 and 4). Similarly, Kotadiya [26] reported overall agreement of RBPT and STAT with I-ELISA was 93.78% and 91.25%, respectively and concluded that I-ELISA to be a better serological test as compared to RBPT and STAT and it could be advocated for screening of sheep for brucellosis. According to ICAR Annual Report [30], RBPT showed the least relative sensitivity than I-ELISA in both sheep and goats, taking complement fixation test as gold standard and I-ELISA could be used as a validated test for diagnosis of brucellosis in small ruminants. Hence, I-ELISA found to be a better serological test as compared to RBPT and STAT and it could be advocated for screening of small ruminants for brucellosis.

## Conclusion

On the basis of the present study, we conclude that seroprevalence of brucellosis was prevalent in small ruminants of the study area. Seroprevalence of brucellosis was significantly more frequent in sheep as compared to goats. When three serological tests were compared, highest seropositivity was found by RBPT followed by STAT and least by I-ELISA. Sensitivity and specificity of I-ELISA were higher than RBPT and STAT for the detection of *Brucella* antibodies. Thus, I-ELISA is a better serological test as compared to RBPT and STAT and it is advocated for screening and diagnosis of brucellosis in small ruminants. Although, there is a need of further evaluation using serum samples from bacteriological isolation positive animal. Beside this, control and prevention programs should be started at the state and national levels for decreasing the incidence of brucellosis. For this an extension education campaign about risk factors of disease, economic, and zoonotic importance of disease should be stated particularly in the high-risk areas, among veterinary practitioners and livestock owners and regular sero-surveillance of the disease needed to know the status of control and prevention programs.

## Authors’ Contributions

The present study was a part of DBS’s original research work during his M.V.Sc thesis program. HHP conceptualized the aim of the study, designed, planned and supervised the experiment and corrected the manuscript. Collection of samples, execution of the experimental study, collation and analysis of data, interpretation of the results, and drafting the manuscript was done by DBS. HCC, VLP, HBP and DRS helped in analyses, draft, and revision of the manuscript. All authors read and approved the final manuscript.
